# The Cross-Cultural Adaptation and Validation of the Polish Version of the Expanded Nursing Stress Scale Tool

**DOI:** 10.1155/2023/9754344

**Published:** 2023-09-05

**Authors:** Natalia Pawlak, Lena Serafin, Bożena Czarkowska–Pączek

**Affiliations:** ^1^Department of Clinical Nursing, Faculty of Health Sciences, Medical University of Warsaw, 27 Erazma Ciolka 27 Street, Warsaw 01-445, Poland; ^2^Doctoral School, Medical University of Warsaw, 81 Zwirki I Wigury Street, Warsaw 02-091, Poland

## Abstract

**Background:**

Nursing is considered one of the most stressful occupational groups. Work-related stress is a major health risk for workers worldwide. Therefore, it is critical to continually monitor nurses' stress levels based on important aspects of nurses' work identified as stressors.

**Methods:**

A total of 331 nurses participated. The cross-cultural adaptation of the instrument was carried out based on six of the seven steps proposed: forward translation of ENSS, comparison of the two translated versions, blind back translation, comparison of the two back-translated versions of ENSS, pilot testing of the prefinal version of ENSS, and full-psychometric testing of the prefinal version of the translated instrument. The reliability of the scales was estimated using Cronbach's alpha and McDonald's omega internal consistency coefficients.

**Results:**

The results indicate that each of the distinguished subscales is unique, but at the same time, they are all related to each other as different dimensions of stress.

**Conclusions:**

The Polish version of the ENSS tool is a valid and reliable tool for assessing the level of stress experienced by nurses.

## 1. Background

Work-related stress is defined as harmful physical and emotional responses that occur when the requirements of a job do not match the capabilities, resources, or needs of the worker [1]. It is also recognized as the response people may have when presented with work demands and pressures that are not matched to their knowledge and abilities and that challenge their ability to cope [2]. Work-related stress is one of the most important workplace health risks for employees worldwide [3]. Nursing is known as one of the most stressful occupational groups [4]. This demanding profession includes many stressors, which are mainly connected with the work environment (workload, shift work, insufficient human resources, low organizational support and empowerment, low salaries, poor interpersonal relationships, burden of emotional labor, and aggression in the workplace) and personal resources (psychological capital, positive affectivity, resilience, hardiness, self-regulation, sensitivity, and affective, depressive, and cyclothymic temperament) [5]. The consequences of occupational stress in the nursing profession are identified as the job's impact on general health and healthy behavior, general psychological health, including depressive and anxiety symptoms, insomnia, burnout, psychosomatic symptoms, and affective and behavioral responses to the work, such as job satisfaction, intent to leave, work engagement, caring behavior, moral distress, and sickness absence [5–8]. The significance of stress for nursing practice is reflected in both nurses' work and the outcomes of their work, as well as in enormous costs incurred by healthcare systems. Therefore, it is crucial to continuously monitor the stress level of nurses based on important aspects of their work that are identified as stressors. Today's nursing work environment is affected by the COVID-19 pandemic experience and specialists' prognosis of the inevitability of future pandemics [9] and is characterized by increasing healthcare complexity and patient demands—all of which contribute significantly to stress experiences. Therefore, monitoring the occurrence of stress among nurses is more important than ever before. This study has undertaken the validation and Polish adaptation of an Expanded Nursing Stress Scale that includes nine aspects of nursing stress in an effort to provide Polish nursing managers and researchers with a comprehensive instrument to evaluate nurses' work-related stress. The ENSS tool is a questionnaire that examines stress, with the advantage that it is dedicated specifically to professional nurses. The validation carried out and presented has cross-cultural and international relevance, as it indicates its universality of use and shows results comparable to other multicultural studies.

Therefore, the study aimed to evaluate the validity and reliability of the Expanded Nursing Stress Scale tool on a sample of Polish nurses.

## 2. Materials and Methods

### 2.1. Design and Sample

This psychometric study used a cross-sectional design. The data were collected in the last quarter of 2021 by one of the researchers using an online tool. The link to the study survey has been presented in different nursing forums and social media groups that engage Polish nurses. Gathering responses through online questionnaires is an established method in healthcare research. The data collection process is simple and fast, ensuring more complete data [10, 11].

Convenience sampling was used in this study, which included 331 participants. For the conducted factor analyses, the researchers indicate samples of 200 and more respondents (based on the justification of the test power) [12–17], and for 300 people, it is considered good in the Clark and Watson [18] and Comrey and Lee study [19]. A calculator for structural equation models (SEMs) [20] was used to calculate the minimum sample size. Assuming the original structure of the questionnaire (9 factors, 57 items) and a moderate effect (0.3), a test was conducted with a power of 0.8 and a significance level of *α* = 0.05. The minimum sample size for the model was 256 individuals. Increasing the test power to 0.95, the minimum sample size (recommended) is 264 [20]. The personal information form contained items related to the sociodemographics of the participants, such as gender, hospital reference level, ward, age, work experience, type of employment, working hours, and professional qualifications. The inclusion criteria for the study were nurses who were professionally active and had a length of service of more than three months. The exclusion criteria were nurses not working directly in patient care.

The cross-cultural adaptation of the instrument was carried out on the basis of six of the seven steps proposed by Sousa and Rojjanasrirat [21]: (1) forward translation of ENSS, (2) comparison of the two translated versions, (3) blind back translation, (4) comparison of the two back-translated versions of ENSS, (5) pilot testing of the prefinal version of ENSS, and (6) full-psychometric testing of the prefinal version of the translated instrument [21]. The step “preliminary psychometric testing of the prefinal version of the translated instrument with a bilingual sample” was excluded from the process due to studied sample characteristics.

### 2.2. Instrument

The Expanded Nursing Stress Scale (ENSS), developed by French et al. [22], is dedicated to measuring the sources and frequency of stress experienced by nurses. ENSS includes a 57-item, nine-factor (death and dying, conflict with physicians, inadequate emotional preparation, problems related to peers, problems related to supervisors, workload, uncertainty concerning treatment, patients and their families, and discrimination), four-point Likert scale with scores ranging from 0 (not encountered) to 4 (always stressful), whereas a score of 5 indicated that it was not applicable. The ENSS Cronbach's alpha coefficients range from 0.74 to 0.88, indicating a good relationship among the measured variables [22].

### 2.3. Translation and Cultural Adaptation of the Instrument

Permission was obtained from the authors of the original scale [22] to utilize the ENSS and conduct translation and cultural adaptation. According to Sousa and Rojjanasrirat [21], four steps have been conducted to translate the ENSS. Step 1 Forward translation: the ENSS scale has been translated into Polish by two people associated with medical sciences and who were fluent in English and Polish. Step 2 Comparison of the two translated versions: researchers assessed version compliance and held a discussion to select the final version. Step 3 Blind back translation: the translation was conducted by two independent translators who were unfamiliar with the original version of the scale and had experience in medical science. Step 4 Comparison of the two back-translated versions: the two translated versions were compared, and the similarities and possible differences were discussed by an expert panel that included researchers and translators. Step 5 Pilot testing of the prefinal version of ENSS: pilot testing and face validity were conducted among 25 nurses using convenience sampling. They did not have any comments about the questionnaire. The time required to complete the questionnaire was ∼15 minutes.

Habits of linguistic expression differ depending on the cultural context [22]. Therefore, to prevent spelling and grammatical errors, the finalized Polish translation was proofread by the researchers.

### 2.4. Psychometric Testing

The last step (6) was established using the initial full-psychometric properties of the newly translated, adapted, and cross-validated instrument with a sample of the target population. Psychometric testing of the ENSS Polish version (ENSS-Pl) has been conducted through (a) content validity assessment, (b) construct validity assessment, and (c) reliability assessment.

#### 2.4.1. Content Validity

Content validity was checked by 10 healthcare workers (nurse managers and nurses from surgical, internal, intensive care, and primary care units). Based on their professionalism and experience in clinical practice, they assessed each item of the scale in terms of clarity and cultural relevance of statements. VREP—validation rubric for expert panels—was used for content validity assessment. Nurses were asked to rate the accuracy of each item on a four-point scale, from 1 (not acceptable—major adjustments needed) to 4 (exceeds expectations—no change required). The content validity index (CVI) was calculated based on the VREP results. According to Polit and Beck, the acceptable CVI values should be at least 0.75. The CVI of each item was calculated by dividing the number of experts who rated it 3 or 4 by the total number of experts [23].

#### 2.4.2. Construct Validity

The scale was evaluated for construct validity using confirmatory factor analysis (CFA). The Kaiser–Meyer–Olkin (KMO) measure was used to test the sample's adequacy. To test the goodness of fit, this study used chi-square/degrees of freedom (*χ*^2^/d*f*), the comparative fit index (CFI), the root mean square error of approximation (RMSEA), the standardized root mean squared residual (SRMR), and the Tucker–Lewis index (TLI). A *χ*^2^ test >0.05 is desirable, although when a large sample is used, the *χ*^2^ test is often significant, and researchers therefore recommend using RMSEA (<0.08 has been reported as acceptable and values <0.06 as good) [24]. The acceptable CFI threshold value and the TLI value were assumed to be 0.9 [25, 26]. Then, due to the lack of confirmation of the structure of the original tool in the Polish adaptation, an exploratory factor analysis (EFA) was carried out using the principal components method with varimax rotation. A value of >0.6 for KMO is considered good [23]. Then, to confirm the result, a Horn analysis was performed.

Construct-related validity, such as convergent and discriminant validity, was examined using Pearson's correlation coefficient and evaluated using the guidelines established by Evans (correlation levels: negligible = 0.00–0.19, weak = 0.20–0.39, moderate = 0.40–0.59, strong = 0.60–0.79, and very strong = 0.80–1.00) [27].

#### 2.4.3. Reliability

The internal consistency of ENSS-Pl subscales has been evaluated using Cronbach's alpha and McDonald's omega coefficients. Values >0.70 have been assessed as a satisfactory result [28].

### 2.5. Statistical Analysis

The analyses were carried out in IBM SPSS Statistics 26 and R.3.6. In the first part, confirmatory factor analysis of the ENSS was performed based on the original structure of the questionnaire. Then, exploratory factor analysis was performed using the principal components method with varimax rotation. The Kaiser criterion and Horn's parallel analysis were used in determining the number of factors. The minimum value of the factor load was assumed to be 0.3. Convergent validity and discriminant validity have been examined by the Pearson correlation coefficient analysis of relationships among individual ENSS factors. The reliability of the scales was estimated using Cronbach's alpha and McDonald's omega internal consistency coefficients. Descriptive statistics were used to describe and characterize the study sample. The data were of normal distribution according to the Kolmogorov–Smirnov test.

The level of significance was *α* = 0.05.

## 3. Results

### 3.1. Participants' Characteristics

A total of 331 nurses completed the survey in this study. Most respondents were female. The study group consisted of nurses aged 22–64 (*M* = 40.40; SD = 10.76) with work experience ranging from 0.25 to 45 years. Most of the respondents (39.3%) were nurses working in a hospital with the third level of reference. Participants' social and demographic information is presented in Table 1.

### 3.2. Psychometric Analyses

#### 3.2.1. Content Validity

CVI was used to assess the content validity of the scale based on the VREP results. The CVI of the items ranged from 0.9 to 1, and the scale CVI was 0.99. Based on the results, none of the items were excluded from the further validation process.

#### 3.2.2. Construct Validity

In the next phase of instrument validation, CFA was performed. The analysis did not confirm the good fit of the data to the model (Table 2).

Therefore, in the further part of the investigation, an exploratory factor analysis was undertaken to establish the ENSS-Pl structure. This analysis was performed using the principal components method with varimax rotation.

**Figure 1:**
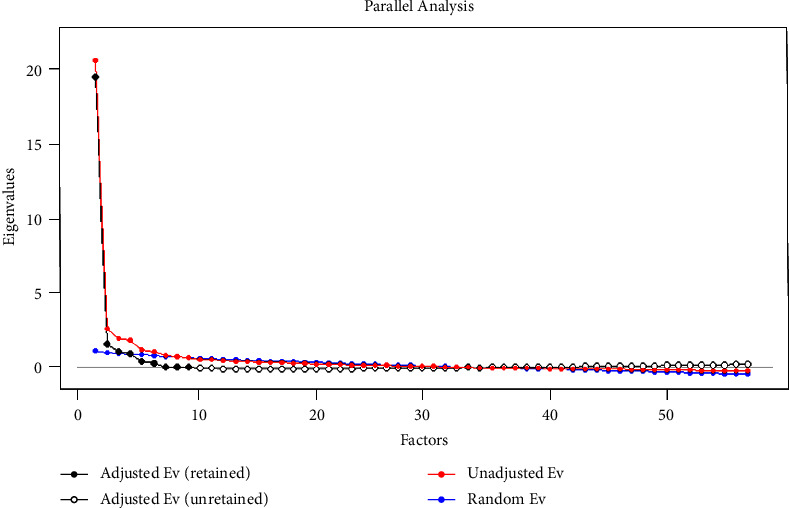
Scree plot.

The application of factorial analysis was considered appropriate, with the Kaiser–Meyer–Olkin (KMO) index = 0.95 and Bartlett's test of sphericity being significant (*χ*^2^ = 11721.57; *df* = 1596; *p* < 0.001). Eight factors were distinguished based on the eigenvalues. The eight-factor solution was also confirmed by Horn's parallel analysis (Figure 1). All the distinguished factors except for nine (professional problems) were characterized by a satisfactory level of reliability (above 0.7). The ninth factor was characterized by a low level of reliability (*α* = 0.39); therefore, we excluded it from the final version of the questionnaire—it contained only two test items.

The rotated component matrix (Table 3) showed the factor loads were greater than 0.36 (0.35 is considered acceptable) [29].

Construct validity has also been evaluated by testing the intercorrelation of the eight factors.  Table 4 presents the Pearson correlation matrix for ENSS-Pl subscales. All analyzed correlation coefficients were positive at a moderate or strong level, from 0.54 to 0.74. These results indicate that each of the distinguished subscales is unique, but at the same time, they are all related to each other as different dimensions of stress.

#### 3.2.3. Internal Consistency

Cronbach's alpha coefficients were found to range from 0.729 to 0.928. McDonald's omega ratio ranged from 0.74 to 0.93. Table 3 shows the factor loads and reliability coefficients of the ENSS-Pl subscales.

## 4. Discussion

Tool validation plays a key role in the field of translating health-related quality-of-life measures and other patient-reported outcome tools [30, 31]. Cross-cultural adaptation of tools helps achieve equivalence between the original source and target versions of the questionnaire [30–32]. It is recognized that for a tool to be used in different countries, it must be not only linguistically well-translated but also culturally adapted to maintain the validity of the instrument's content at the conceptual level across cultures [31, 32]. Attention to this level of detail increases confidence that the impact of the instrument is described in a similar way in international studies or outcome evaluations [30, 32]. This process will ensure that psychometric properties such as relevance and reliability are maintained at the expected level [30, 32].

The two scales used most in the literature to measure stress in the nursing population are the Nursing Stress Scale (NSS) and the Perceived Stress Scale (PSS-10). As a result of several surveys of nurses, a need for an NSS tool with expanded questions for respondents was noted. Therefore, the update was developed and named “ENSS.” The authors' goal was to develop a reliable and valid measure based on the research process and their theoretical understanding of stress [33]. In conclusion, to make an in-depth analysis of the causes of stress among nurses, the ENSS scale, which included the five subscales of the NSS scale, and the added two subscales allow for a more reliable analysis and interpretation of the results [34]. This scale can measure the reaction to an event. The scale contains 10 questions. The five-point scale (0 = never and 4 = very often), in a similar way to the ENSS, allows for assessing stress among nurses. What the ENSS and PSS-10 scales have in common is that they are used to assess the intensity of stress related to one's own life situation. What differs between the two scales is that the PSS-10 can also be used as a form of interview [34], but it has a narrow time frame that the respondent can be asked about. It provides only a month's perspective. The multidimensionality of the ENSS tool is very timely due to the possibility that emotional manifestations of stress may also appear, depending on the nurses' ability to perceive and control events. Psychological differences affect an individual's response to a stressful event, so predicting based on the situation alone is quite a difficult task. ENSS provides the opportunity to study this phenomenon as it considers the assessment of stress between the individual and the individual's work environment.

The analysis was conducted according to Sousa and Rojjanasrirat [21]. The translation procedure used in the study guaranteed semantic equivalence and cultural matching. The translators acted fully independently in order to avoid misconceptions and reproduction. In addition, the panel of experts confirmed the comprehensibility of the questions in the questionnaire, their adequacy, and their full-applicability among nurses.

The analysis shows that the ENSS-Pl questionnaire required modifications to the subscales. Changes were made to the number of subscales and the number of questions in each subscale. The original tool included nine subscales: death and dying, conflict with doctors, inadequate emotional preparation, problems with peers, problems with superiors, workload, uncertainty about treatment, patients and their families, and discrimination. In ENSS-Pl, there were eight subscales: death and dying, conflict with physicians and supervisors, inadequate optional preparation, problems with patients and their families, workload, uncertainty concerning treatment, patients and their families, and discrimination. Two questions were rejected due to low reliability (viz., performing procedures that patients experience as painful and criticism by a physician). The analysis clearly showed that the Polish adaptation of the ENSS tool used 55 research questions, each of the highlighted subscales was unique, and together they explored different dimensions of stress. (see Table S1 in Supplementary Material for a comprehensive analysis). Finally, the adapted 55-item ENSS-Pl showed adequate internal reliability in all domains, with Cronbach's alpha and McDonald's omega values reaching an acceptable score, indicating a good relationship among the measured variables. This result is consistent with the reliability results of the original ENSS [35]. All analyzed correlation coefficients were positive at a moderate or strong level. Our validation analysis showed a fair assessment of the scale's clarity and relevance. As stated by the authors of the original ENSS scale, it is designed to predict nurses' stress in various healthcare settings [35].

The adaptation of the questionnaire to Polish conditions is crucial because there is no tool in Poland that comprehensively examines this phenomenon on such different levels. Each of the scales of the ENSS tool covers one or more sources of stress, and more than that, the results of the ENSS authors' study and this study clearly show that this is a significant problem for nurses; it is worth using this tool in the future to monitor stress issues.

Using the same tool in different countries makes it possible to analyze the magnitude of the phenomenon globally through data syntheses, which provide the highest-powered scientific evidence based on which interventions are planned to implement changes relevant to nursing development.

## 5. Limitations

Several limitations of the survey should be noted. Relatively few men participated, which may limit the representativeness of the sample and the generalizability of the results. Nevertheless, the total sample size meets the requirements. The tool for assessing stress among active-duty nurses could be made more valid by retesting the instrument on a larger number of respondents to identify and remove areas that are redundant, thereby improving the tool's potential. The depth and breadth of data collected in this study provide researchers with an initial level of comfort in using the ENSS-Pl scale, which provides a basis for further investigation in the healthcare sector. Despite these limitations, our study shows that the Polish version of the ENSS-Pl is a reliable and valid tool that can be used to assess stress levels among nurses.

## 6. Conclusions

This cross-cultural adaptation of the ENSS-Pl for nurses confirms that it is a tool with potential for use in different geographic areas, including Poland. Since ENSS has been adopted in many countries, ENSS-Pl will allow Poland to take part in global research aimed at ensuring appropriate and friendly work environments. Sharing global experiences in this area is of great importance due to the low number of nurses worldwide and high job turnover, and it could also help reverse these negative trends.

## 7. Implications for Nursing Management

Stress and coping strategies in clinical situations are of interest to nurses. To cope, it is important to assess and understand the team's resilience to stressors. The ENSS-Pl validated in this study will help analyze nurses' stress for Polish healthcare managers. This assessment will help create specific interventions to improve the quality of life of the nursing team.

## Figures and Tables

**Table 1:** Social and demographic information of the participants (*n* = 331).

Descriptive characteristics	Percentage (frequency)	(*M* ± SD)
Sex		
Female	92.7% (307)	
Male	7.3% (24)	
Age		40.40 ± 10.8
Professional experience (year)		14.56 ± 12.6
Professional experience in the current unit		9.70 ± 10.4
Hospital		
I reference level	24.8% (82)	
II reference level	21.5% (71)	
III reference level	39.3% (130)	
Other	14.4% (48)	
Type of employment		
Full-time	92.4% (306)	
Contract	4.8% (16)	
Civil contract	2.7% (9)	
Working hours		
8-hour system	16.0% (53)	
12-hour system	77.6% (257)	
24-hour system	3.9% (13)	
Flexible working hours	2.2% (7)	
No data	0.3% (1)	
Professional qualifications		
Medical high school	6.9% (23)	
Bachelor of nursing	42.9% (142)	
Master of nursing	49.8% (165)	
Doctor (PhD)	0.3% (1)	
Specialization training (number of training sessions)		2.03 ± 3.85

**Table 2:** Summary of CFA match indicators.

Models	d*f*	*χ* ^2^	*χ* ^2^/d*f*	*p*	CFI	RMSEA	SRMR	TLI
ENSS	1503	4080.62	2.71	<0.001	0.763	0.072	0.070	0.748
Hu and Bentler [25]			<5.0	>0.05	≥0.95	<0.08	<0.08	≥0.95

CFI, comparative fit index; RMSEA, root mean square error of approximation; SRMR, standardized root mean square residual; TLI, Tucker–Lewis index.

**Table 3:** Descriptive statistics, reliability coefficients, and factor loads of the ENSS (*n* = 331).

Factor names	Items	Factors loads	% of explained variance	Cronbach's alpha [95% CI]	McDonald's omega [95% CI]
Death and dying	5-items		4.13	0.83 [0.82; 0.84]	0.84 [0.80; 0.87]
	Item-9	0.59			
	Item-17	0.60			
	Item-27	0.76			
	Item-37	0.59			
	Item-53	0.65			

Conflict with physicians and supervisors	8-items		3.94	0.85 [0.84; 0.87]	0.88 [0.86; 0.90]
	Item-5	0.47			
	Item-6	0.45			
	Item-10	0.68			
	Item-13	0.36			
	Item-14	0.69			
	Item-24	0.58			
	Item-25	0.54			
	Item-28	0.45			

Inadequate optional preparation	4-items		2.06	0.73 [0.71; 0.75]	0.74 [0.68; 0.78]
	Item-3	0.53			
	Item-4	0.64			
	Item-12	0.55			
	Item-23	0.47			

Problems with patients and families	4-items		2.82	0.80 [0.79; 0.82]	0.81 [0.76; 0.85]
	Item-7	0.75			
	Item-15	0.69			
	Item-35	0.51			
	Item-44	0.62			

Workload	16-items		37.09	0.93 [0.92; 0.93]	0.93 [0.92; 0.94]
	Item-21	0.47			
	Item-29	0.41			
	Item-30	0.67			
	Item-31	0.62			
	Item-32	0.46			
	Item-36	0.50			
	Item-38	0.37			
	Item-39	0.63			
	Item-40	0.77			
	Item-41	0.58			
	Item-42	0.59			
	Item-43	0.50			
	Item-46	0.51			
	Item-49	0.60			
	Item-54	0.70			
	Item-55	0.44			

Uncertainty concerning treatment	5-items		2.59	0.80 [0.79; 0.82]	0.81 [0.77; 0.84]
	Item-11	0.47			
	Item-18	0.59			
	Item-19	0.47			
	Item-56	0.56			
	Item-57	0.55			

Patients and their families	3-items		2.01	0.78 [0.76; 0.79]	0.78 [0.73; 0.82]
	Item-33	0.55			
	Item-34	0.57			
	Item-45	0.54			

Discrimination	10-items		5.23	0.89 [0.89; 0.90]	0.91 [0.89; 0.92]
	Item-8	0.67			
	Item-16	0.76			
	Item-20	0.51			
	Item-22	0.54			
	Item-26	0.72			
	Item-47	0.43			
	Item-48	0.50			
	Item-50	0.72			
	Item-51	0.57			
	Item-52	0.64			

**Table 4:** Correlation matrix between the distinguished factors.

	1	2	3	4	5	6	7	8
Factor 1	1							
Factor 2	0.66	1						
Factor 3	0.63	0.58	1					
Factor 4	0.74	0.69	0.61	1				
Factor 5	0.65	0.60	0.54	0.68	1			
Factor 6	0.72	0.60	0.65	0.70	0.61	1		
Factor 7	0.59	0.58	0.54	0.65	0.67	0.61	1	
Factor 8	0.66	0.62	0.61	0.54	0.59	0.60	0.55	1

## Data Availability

The data used in this study are available from the corresponding author upon reasonable request.

## Supplementary Materials

Supplementary Materials Table S1 presents a comparison between the ENSS-Pl questionnaire and the original ENSS questionnaire. Adjustments were introduced in terms of subscale quantity and the number of questions within each subscale. The initial version had nine subscales, whereas ENSS-Pl featured eight. Two questions were excluded due to insufficient reliability, specifically those related to performing procedures perceived as painful by patients and facing criticism from physicians. The analysis distinctly indicated that the Polish version of the ENSS tool employed a total of 55 research queries. Notably, each emphasized subscale possessed its distinctiveness, collectively delving into various stress-related dimensions.
