# Supplementary material for: The Cross-Cultural Adaptation and Validation of the Polish Version of the Expanded Nursing Stress Scale Tool
Source: J Nurs Manag. 2023 Sep 5;2023:9754344. doi: 10.1155/2023/9754344 (PMC11919138; doi:10.1155/2023/9754344)
Supplement: Supplementary Materials — Table S1 presents a comparison between the ENSS-Pl questionnaire and the original ENSS questionnaire. Adjustments were introduced in terms of subscale quantity and the number of questions within each subscale. The initial version had nine subscales, whereas ENSS-Pl featured eight. Two questions were excluded due to insufficient reliability, specifically those related to performing procedures perceived as painful by patients and facing criticism from physicians. The analysis distinctly indicated that the Polish version of the ENSS tool employed a total of 55 research queries. Notably, each emphasized subscale possessed its distinctiveness, collectively delving into various stress-related dimensions. [file 9754344.f1.docx]

Table S1 presents a comparison between the ENSS-Pl questionnaire and the original ENSS questionnaire. Adjustments were introduced in terms of subscale quantity and the number of questions within each subscale. The initial version had nine subscales, whereas ENSS-Pl featured eight. Two questions were excluded due to insufficient reliability, specifically those related to performing procedures perceived as painful by patients and facing criticism from physicians. The analysis distinctly indicated that the Polish version of the ENSS tool employed a total of 55 research queries. Notably, each emphasized subscale possessed its distinctiveness, collectively delving into various stress-related dimensions.

**Table S1.** Factors and questions described based on ENSS-Pl Version and the Corresponding Original Names.

| Factor | ENSS-Pl | Factor | Original ENSS version |
| --- | --- | --- | --- |
| **1.Death and dying** | Feeling helpless in the case of a patient who fails to improve. (#7)  Listening or talking to a patient about his/her approaching death.(#15)  The death of a patient. (#25)  The death of a patient with whom you have developed a close relationship. (# 35)  Watching a patient suffer. (# 51) | **1.Death and dying** | Performing procedures that patients experience as painful. (#1)  Feeling helpless in the case of a patient who fails to improve. (#9)  Listening or talking to a patient about his/her approaching death. (#17)  The death of a patient. (#27)  The death of a patient with whom you have developed a close relationship. (#37)  Physician not being present when a patient dies. (#47)  Watching a patient suffer. (#53) |
| **2.Conflict with physician and supervisors** | Conflict with a supervisor. (#3)  Inadequate information from a physician regarding the medical condition of a patient. (#4)  Conflict with a physician. (#18)  Unpredictable staffing and scheduling. (#11)  A physician ordering what appears to be inappropriate treatment for a patient. (#12)  A physician not being present in a medical emergency. (#22)  Being blamed for anything that goes wrong.(#23)  Disagreement concerning the treatment of a patient. (#26) | **2.Conflict with physicians** | Criticism by a physician. (# 2)  Conflict with a physician. (#10)  Disagreement concerning the treatment of a patient. (#28)  Making a decision concerning a patient when the physician is unavailable. (#38)  Having to organize physicians’ work. (#48) |
| 3.**Inadequate optional preparation** | Feeling inadequately prepared to help with the emotional needs of a patient’s family. (#1)  Lack of an opportunity to talk openly with other unit personnel about problems in the work setting. (#2)  Lack of an opportunity to share experiences and feelings with other personnel in the work setting. (#10)  Not enough time to provide emotional support to a patient. (#21) | **3.Inadequate emotional preparation** | Feeling inadequately prepared to help with the emotional needs of a patient’s family. (#3)  Being asked a question by a patient for which I do not have a satisfactory answer. (#11)  Feeling inadequately prepared to help with the emotional needs of a patient. (#19) |
| 4.**Problems with patients and their families** | Patients making unreasonable demands. (#5)  Patients’ families making unreasonable demands. (#13)  Having to deal with violent patients. (#33)  Having to deal with abusive patients. (#42) | **4.Problems relating to peers** | Lack of an opportunity to talk openly with other unit personnel about problems in the work setting. (#4)  Lack of an opportunity to share experiences and feelings with other personnel in the work setting. (#12)  Lack of an opportunity to express to other personnel on the unit my negative feelings toward patients. (#20)  Difficulty in working with a particular nurse (or nurses) outside my immediate work setting. (#22)  Difficulty in working with a particular nurse (or nurses) in my immediate work setting. (#21)  Difficulty in working with nurses of the opposite sex. (#50) |
|  |  | **5.Problems relating to supervisors** | Conflict with a supervisor. (#5)  Lack of support from my immediate supervisor. (#30)  Lack of support by nursing administrators. (#40)  Lack of support by other health care administrators. (#49)  Criticism by a supervisor. (#31)  Being held accountable for things over which I have no control. (#46)  Criticism from nursing administration. (#54) |
| **5.Work Load** | Difficulty in working with a particular nurse (or nurses) in my immediate work setting. (#19)  Feeling in adequately trained for what I have to do. (#27)  Lack of support from my immediate supervisor. (#28)  Criticism by a supervisor. (#29)  Not enough time to complete all of my nursing tasks. (#30)  Being exposed to health and safety hazards. (#34)  Making a decision concerning a patient when the physician is unavailable. (#36)  Being in charge with inadequate experience (#37)  Lack of support by nursing administrators. (#38)  Too many non-nursing tasks required such as clerical work. (#39)  Not enough staff to adequately cover the unit. (#40)  Uncertainty regarding the operation and functioning of specialized equipment. (#41)  Being held accountable for things over which I have no control. (#44)  Lack of support by other health care administrators. (#47)  Criticism from nursing administration. (#52)  Having to work through breaks. (#53) | **6.Work Load** | Unpredictable staffing and scheduling. (#13)  Too many non-nursing tasks required such as clerical work. (#41)  Not enough time to provide emotional support to a patient. (#23)  Not enough time to complete all of my nursing tasks. (#32)  Not enough staff to adequately cover the unit. (#42)  Not having enough time to respond to the needs of the patients’ families. (#45)  Demands of patient classification system. (#51)  Having to work through breaks. (#55)  Having to make decisions under pressure. (#57) |
| **6.Uncertainty concerning treatment** | Being asked a question by a patient for which I do not have a satisfactory answer. (#9)  Fear of making a mistake in treating a patient. (#16)  Feeling inadequately prepared to help with the emotional needs of a patient. (#17)  Not knowing whether patients’ families will report you for inadequate care. (#54)  Having to make decisions under pressure. (#55) | **7.Uncertainty concerning treatment** | Inadequate information from a physician regarding the medical condition of a patient. (#6)  A physician ordering what appears to be inappropriate treatment for a patient. (#14)  Fear of making a mistake in treating a patient. (#18)  A physician not being present in a medical emergency. (#24)  Not knowing what a patient or a patient’s family ought to be told about the patient’s condition and its treatment. (#33)  Being exposed to health and safety hazards. (#36)  Uncertainty regarding the operation and functioning of specialized equipment. (#43)  Feeling in adequately trained for what I have to do. (#29)  Being in charge with inadequate experience (#39) |
| **7.Patients and their families** | Not knowing what a patient or a patient’s family ought to be told about the patient’s condition and its treatment. (#31)  Being the one who has to deal with patients’ families. (#32)  Not having enough time to respond to the needs of the patients’ families. (#43) | **8.Patients and their families** | Patients making unreasonable demands. (#7)  Patients’ families making unreasonable demands. (#15)  Being blamed for anything that goes wrong. (#25)  Being the one who has to deal with patients’ families. (#34)  Having to deal with violent patients. (#35)  Having to deal with abusive patients. (#44)  Having to deal with abuse from patients’ families. (#52)  Not knowing whether patients’ families will report you for inadequate care. (#56) |
| **8.Discrimination** | Being sexually harassed. (#6)  Experiencing discrimination because of race or ethnicity. (#14)  Lack of an opportunity to express to other personnel on the unit my negative feelings toward patients. (#18)  Difficulty in working with a particular nurse (or nurses) outside my immediate work setting. (#20)  Experiencing discrimination on the basis of sex. (#24)  Physician not being present when a patient dies. (#45)  Having to organize physicians’ work. (#46)  Difficulty in working with nurses of the opposite sex. (#48)  Demands of patient classification system.(#49)  Having to deal with abuse from patients’ families. (#50) | **9.Discrimination** | Being sexually harassed. (#8)  Experiencing discrimination because of race or ethnicity. (#16)  Experiencing discrimination on the basis of sex. (#26) |
